# Perceived health facility barriers to HIV linkage and retention in western Kenya

**DOI:** 10.1186/1471-2334-14-S2-P30

**Published:** 2014-05-23

**Authors:** J Wachira, V Naanyu, B Genberg, S Ndege, AM Siika, S Kimayo, J Mamlin, P Braitstein

**Affiliations:** 1 Academic Model Providing Access to Healthcare (AMPATHPlus) Partnership, Eldoret, Kenya; 2 Moi University School of Medicine, Eldoret, Kenya; 3 Moi University School of Public Health, Eldoret, Kenya; 4 Indiana University School of Medicine, Indianapolis, USA; 5 Regenstrief Institute, Inc. Indianapolis, USA; 6 Dalla Lana School of Public Health, University of Toronto, Toronto, Canada; 7 Brown University, Department of Health Services, Policy & Practice, Providence, Rhode Island, USA

## Introduction

HIV linkage and retention rates in sub-Saharan Africa remain low. The objective of this study was to explore perceived health facility barriers to linkage and retention in an HIV care program in western Kenya.

## Methods

This qualitative study was conducted between, July 2012-August 2013. A total of 150 participants including; 59 patients (HIV, TB, and hypertension); 16 caregivers; 10 community leaders; and 65 healthcare workers, were purposely sampled from three Academic Model Providing access to Healthcare (AMPATHplus) sites. We conducted 16 In-depth interviews and 16 focus group discussions (FGDs) in either, English, Swahili, Kalenjin, Teso, or Luo. All data were audio recorded, transcribed, translated to English, and a content analysis performed. Demographic data was only available for those who participated in the FGDs.

## Results

The mean age of participants in the FGDs was 36 years (SD=9.24). The majority (87%) were married, (62.7%) had a secondary education level and above, and (77.6%) had a source of income. Overall, there were no differences in the perceived barriers across all study participants. Salient barriers reflected on patients’ satisfaction with HIV care. There were similarities in some of the barriers cited for linkage and retention including access to health facilities, stigma associated with health facilities, service efficiency, poor provider-patient interactions, and lack of patient incentives. Barriers unique to linkage were reported as, quality of post-test counseling and poor patient reception at the clinics. Obstacles unique to retention were frequency of clinic appointments, different appointments for mother and child, lack of HIV care for institutionalized populations including students and prisoners, lack of food support, and inconsistent linkage data.

## Conclusion

Our findings revealed that there were similarities and differences between perceived barriers to linkage and retention. The cited barriers reflected on the need for a more patient-centered approach to HIV care. We believe that addressing health facility barriers may ultimately be easier than addressing patient related (intrapersonal and interpersonal) barriers.

